# Image guidance system use amongst Canadian otolaryngologists: a nationwide survey

**DOI:** 10.1186/s40463-022-00581-x

**Published:** 2022-06-13

**Authors:** T. McHugh, D. D. Sommer, A. MA ThambooTewfik, K. A. Smith, Tobial McHugh

**Affiliations:** 1 Division of Otolaryngology, Head and Neck Surgery, Department of Surgery, McGill University, Montreal, QC Canada; 2 Division of Otolaryngology, Head and Neck Surgery, Department of Surgery, McMaster University, Hamilton, ON Canada; 3 Division of Otolaryngology, Head and Neck Surgery, Department of Surgery, University of British Columbia, Vancouver, BC Canada; 4 Department of Otolaryngology, Head and Neck Surgery, University of Manitoba, Winnipeg, MB Canada; 5 A.RC.4221 ENT Clinic, The Montreal Children’s Hospital, 1001 Boulevard Décarie, Montréal, QC H4A 3J1 Canada

**Keywords:** Image guidance, Sinus surgery, Survey, Canada

## Abstract

**Background:**

The use of image guidance systems has gained widespread acceptance as an adjunctive tool for endoscopic sinus surgery. However, the accessibility and usage of this technology is variable across hospitals in Canada.

**Study objective:**

The aim of this study is to investigate the availability, usage, and related issues surrounding the use of image guidance systems in endoscopic sinus surgery across Canadian otolaryngology practice settings.

**Methods:**

An online survey was electronically distributed to practicing otolaryngologists across Canada. The survey contained 27 questions pertaining to the availability, usage, barriers and overall experience of image guidance systems.

**Results:**

The survey was electronically sent to a total of 654 Canadian otolaryngologists of which 158 responded (response rate 24.2%). Image guidance was available to 56.3% of respondents. Of the respondents without access to IGS, 85.5% indicated they would use it if it was available. Financial (capital cost) was identified as the most important barrier in obtaining IGS by 76.3% of respondents.

**Conclusion:**

Over half of Canadian otolaryngologists have access to IGS with over 85% of those without access interested in using it if it was made available. A multitude of different factors contribute to this disparity. We hope that the results of this study will help support Canadian otolaryngologists to access IGS.

**Graphical Abstract:**

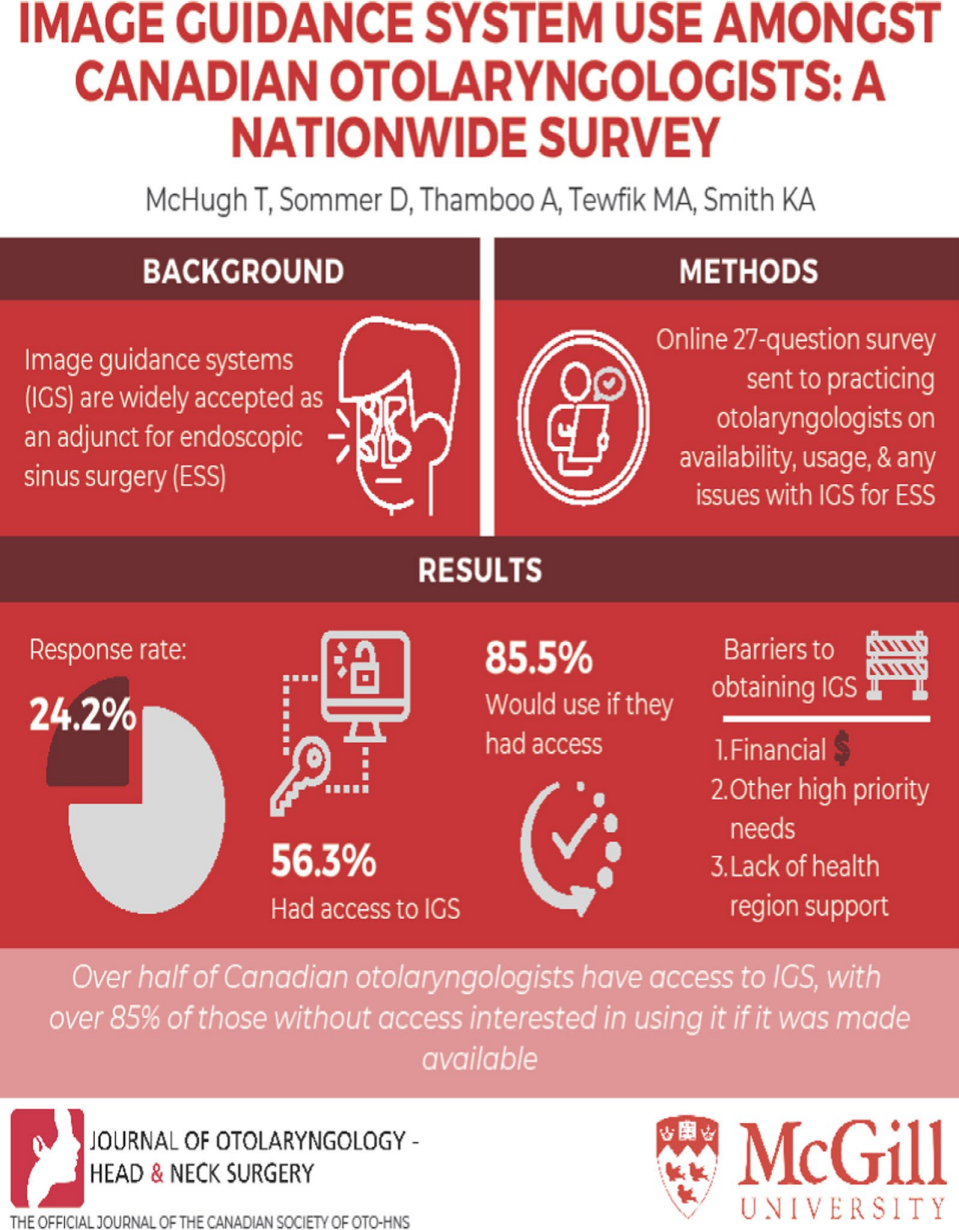

## Introduction

Image guidance systems (IGS) have been available since the 1990s. Over the last decade, there has been a significant increase in the usage of IGS within otolaryngology, especially for endoscopic sinus and skull base surgery [1]. The use of IGS for both primary and revision endoscopic sinus surgery (ESS) is broadly accepted as an effective tool to help navigate potentially complex anatomical relationships and complement standard ESS [2]. IGS ESS is associated with reduced major and total complications when compared to non-IGS ESS [3, 4] and allow for a more complete and thorough operation [5]. The American Academy of Otolaryngology have published a position statement with recommended indications for IGS which include the following: revision sinus surgery; distorted sinus anatomy; extensive sino-nasal polyposis; pathology involving the frontal, posterior ethmoid and sphenoid sinuses; disease abutting the skull base, orbit, optic nerve or carotid artery; cerebrospinal fluid (CSF) rhinorrhea or conditions where there is a skull base defect; benign and malignant sino-nasal neoplasms [6].

Despite these clear guidelines and widespread acceptance of IGS for ESS, there are anecdotally reported regional and institutional disparities regarding the use of IGS across Canada. Such variation may affect the accessibility and equity of surgical care across Canada. Our goal is to quantify the potential disparities to help inform surgeons, institutions, and policy makers of the current variations in IGS access and use in Canada. A better understand of the current landscape of IGS utilization may help support those who aspire to utilize IGS in their practice. The overall aim of this study is to investigate the availability, usage, and issues surrounding the use of IGS in ESS across Canadian otolaryngologist.

## Materials & methods

An online survey was distributed to Canadian otolaryngologists across the country. Provincial regional heads of otolaryngology society groups emailed the online survey to all provincial society members. Responses were collected from February 10th to May 6th, 2021, and included two additional email reminders to complete the survey.

The survey consisted of three parts focusing on clinico-demographics, availability of IGS and overall experience with its use. All questions were created and reviewed by the authors of this study in a interative review process. The survey consisted of a total of 15 or 19 questions depending on the availability of IGS. All questions were mandatory and additional comments could be made if the respondant wished to convey further information. The total survey time was approximately 5–10 min. Consent to participate was outlined in both the email sent to participants as well as the initial page of the survey and was implicit on response. All responses were anonymous, and no personal or identifiable information was recorded. A 3rd party survey website was used for data collection and storage (forms.google.com). Data was exported to Microsoft Excel (Microsoft© v16.50) for analysis and descriptive statistics were used to analyze the data. We collected both quantitative data (scalable questions) and qualitative data (asking open ended questions/comments). Survey responses were reported as percentages. Subgroup analyses were also performed to further investigate provincial disparities and differences between type of practice (community vs. academic). Due to limitations of the data collected, no complex statistical analyses were performed. Institutional ethics review necessity was inquired from McMaster University’s institutional Hamilton Integrated Research Ethics Board (HiREB), and determined to not be required for this study.

## Results

The survey was electronically distributed to a total of 654 otolaryngologists across Canada, of which 158 responded (response rate 24.2%).

### Practice demographics

**Fig. 1:**
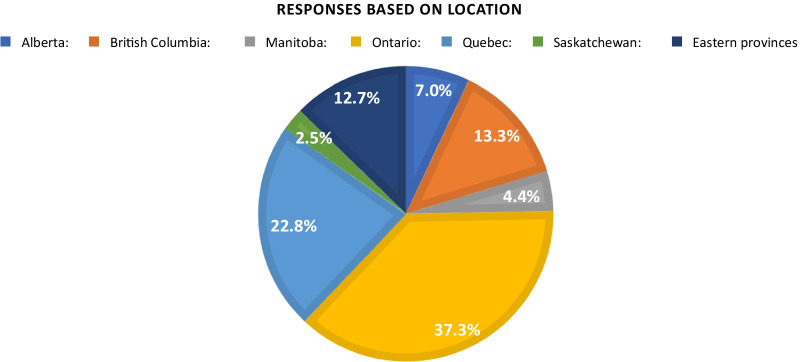
Geographic location of survey respondents

**Fig. 2:**
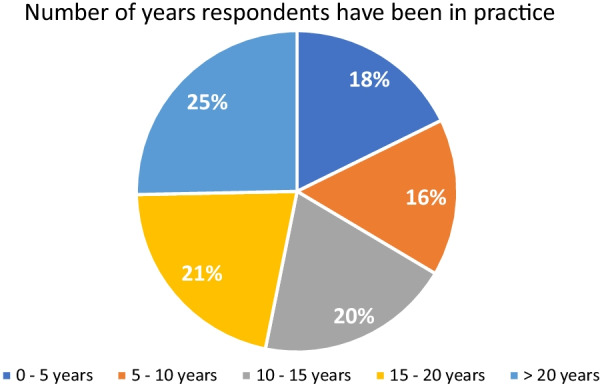
Number of years respondents have been in practice

Responses were received from all provinces in Canada, with the majority coming from Ontario and Quebec (37.3% and 22.8%, respectively) (Fig. 1). The years of practice was distributed relatively evenly among the respondents (Fig. 2); 25.3% of respondents had > 20 years of experience, followed by 15–20 years (21.5%), 10–15 years (19.6%), 5–10 years (15.8%) and 0–5 years (17.7%) of experience. The majority of respondents had a community practice (60.8%) with the remaining divided equally between an academic practice and a mixed practice. The majority of respondents did not have subspecialty training (61.4%). Of those with subspecialty training (38.6%), the majority were in rhinology and skull base (36.1%) followed by head and neck surgery (23.0%), then pediatric otolaryngology (19.7%), facial plastics (14.8%), otology and neurotology (13.1%) and laryngology (9.8%).

**Fig. 3:**
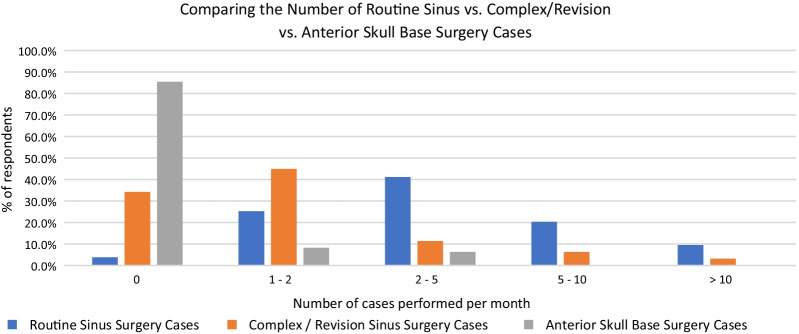
Comparing the number of cases per month respondents are performing routine sinus surgery, complex/revision sinus surgery, and anterior skull base surgery

Respondents were asked to quantify their experience with sinus and skull base surgery. The majority of respondants performed routine sinus surgery regularily (70.9% > 2 cases/month) (Fig. 3). Regarding complex or revision sinus surgeries, 34.2% of respondents do not perform any of these types of surgeries per month, 44.9% perform 1–2 complex/revision cases per month, 11.4% perform 2–5 cases per month, 6.3% perform 5–10 cases per month, and 3.2% perform more than 10 complex/revision cases per month (Fig. 3). Anterior skull base surgery was less commonly performed among respondents; 85.4% of respondents do not perform any anterior skull base surgeries, 8.2% perform 1–2 cases per month and 6.3% perform 2–5 cases per month (Fig. 3).

### Access to IGS

**Fig. 4:**
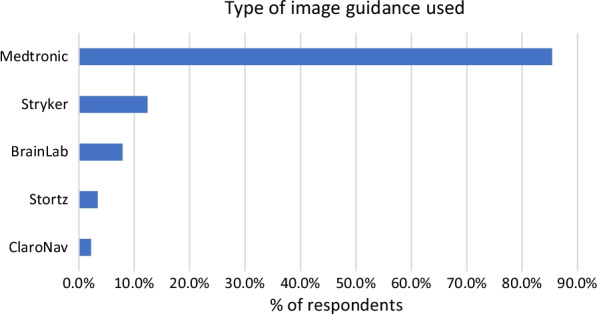
Type of image guidance system used by respondents

**Fig. 5:**
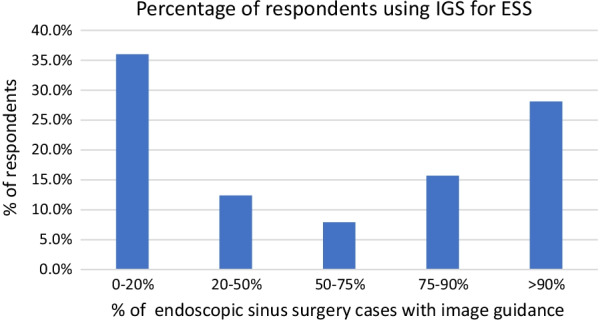
Percentage of endoscopic sinus surgeries respondents are using image guidance systems (not including skull base surgery)

Just over half of the respondents have access to IGS at their hospital (56.3%). Of those with access to IGS, 73.0% had access only one IGS available at their hospital. 21.3% have two systems available, 3.4% have three systems available and 2.2% have more than 5 systems available. The most common IGS available is from Medtronic (84.3%) (Fig. 4). The majority (76.4%) used an electromagnetic IGS and 32.6% used an optical IGS (Fig. 4). The surgical service that is the primary user of IGS was Otolaryngology, Head and Neck surgery (77.5%) followed by Neurosurgery (21.3%) and Orthopaedic/Spine surgery (1.1%). Regarding usage of IGS for ESS (not including skull base surgery), respondents reported using it for almost all cases (28.1% use for > 90% of cases) or for few cases (36.0% use it 0–20% of cases) with the remainder distributed between (Fig. 5).

**Fig. 6:**
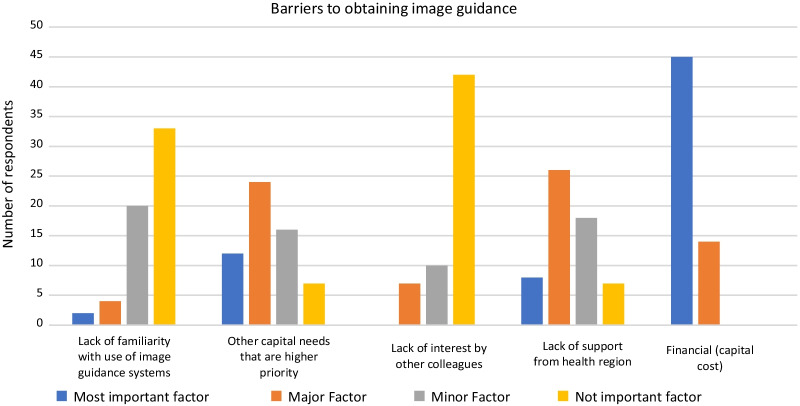
Barriers for obtaining an image guidance system (for respondents without access)

Of the respondents without access to IGS, 85.5% would use it if it was available. Financial (capital cost) was identified as the most important barrier in obtaining IGS by 76.3% of respondents. Other factors such as lack of support from health region and other capital needs that are higher priority were also identified as the most important or major factors (by 57.6% and 61.0% of respondents, respectively) (Fig. 6). A large majority of the respondents (83.1%) without access to IGS mentioned that lack of access to IGS prevented them from performing surgeries that they would otherwise undertake.

**Fig. 7:**
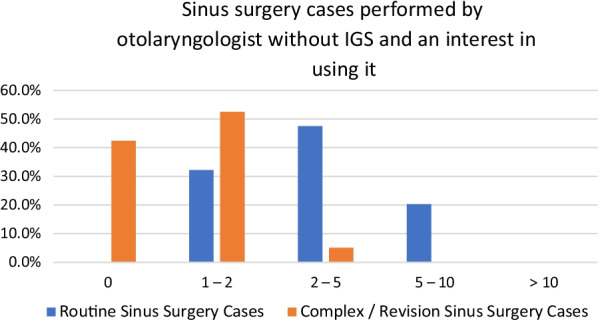
Number of cases per month community otolaryngologist are performing routine sinus surgery, complex/revision sinus surgery, and anterior skull base surgery

To gain more insight about he 85.5% of respondents without IGS access and expressing interest, a subgroup analysis looking at the amount and type of sinus surgergies they perform was assessed. The majority of these respondents mention performing at least two to five routine surgeries per month (47.5%) and one to two complex/revision sinus surgeries per month (54.2%) (Fig. 7).

**Fig. 8:**
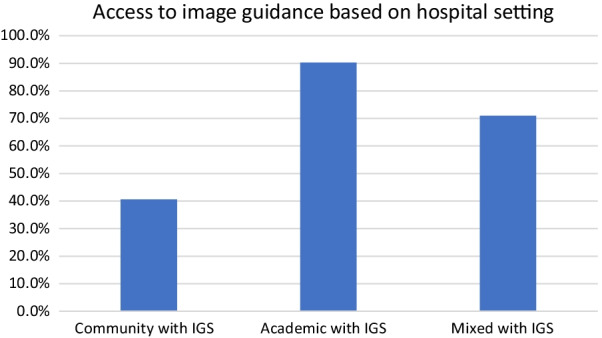
Comparing access to image guidance based on hospital setting/practice type

**Fig. 9:**
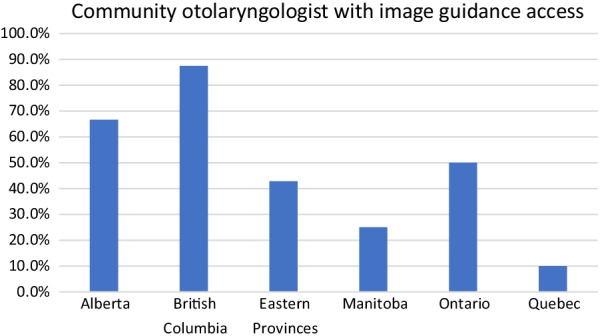
Comparing community otolaryngologist access to image guidance based on province

Another subgroup analysis using the type of practice and access to IGS demonstrated that 40.6% of community otolaryngologist have access to IGS and 71.0% of those working in a mixed practice also have access to IGS. Despite having an academic practice, two respondents from Ontario and one from Quebec mentioned not having IGS access. The rest of the academic otolaryngologists (90.3%) mention having access to IGS (Fig. 8). A provincial breakdown of community otolaryngologist with IGS access revealed that British Columbia have the most access (87.5%) while Quebec have the lowest amount of access (10.0%) (Fig. 9).

### Use of IGS

**Fig. 10:**
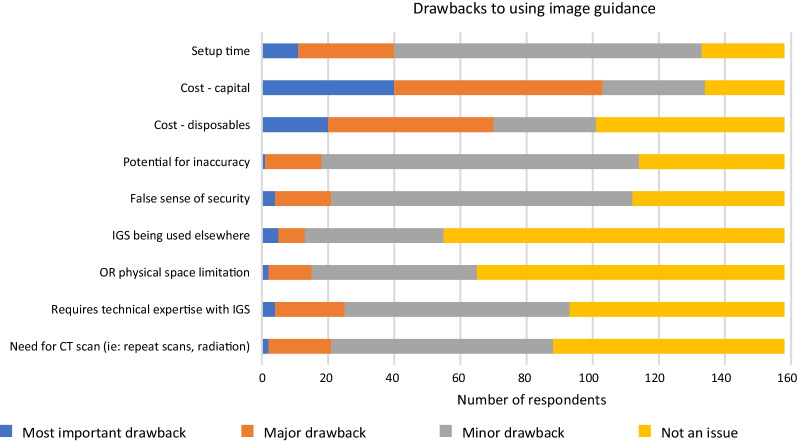
Potential drawbacks identified by all respondents for using image guidance

**Fig. 11:**
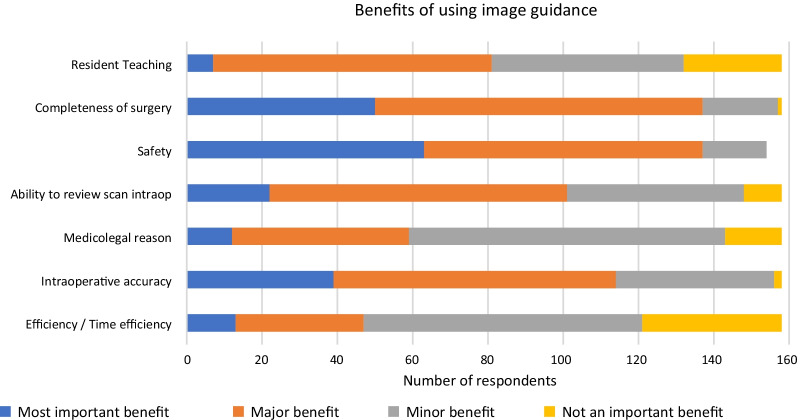
Potential benefits identified by all respondents for using image guidance

Regarding potential drawbacks of using IGS, capital cost and disposable cost were identified as the most important barrier to IGS use (65.2% and 44.3% of respondents, respectively) (Fig. 10). Regarding potential benefits of using IGS, completeness of surgery and safety were identified as the most important benefits of IGS use (86.7% of respondents for each factor, respectively) (Fig. 11).

### Indications for IGS

**Fig. 12:**
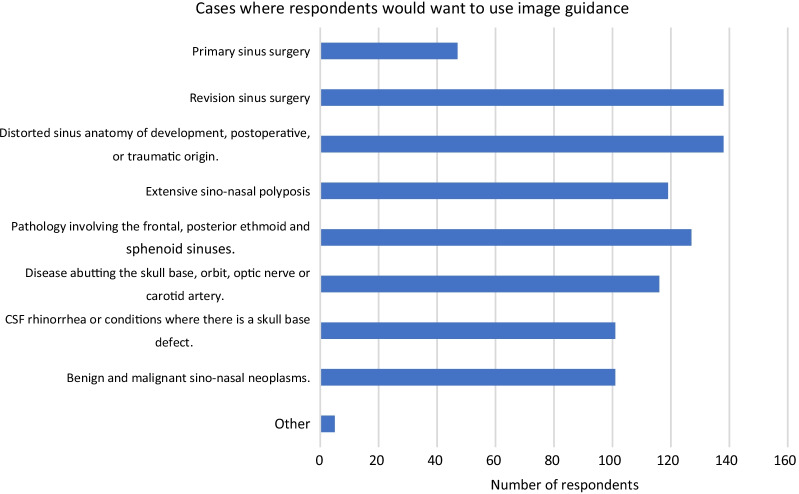
Indications for image guidance systems identified by all respondents

Indications surrounding the use of IGS were also asked. 29.7% of respondents mentioned its indication in primary sinus surgery; 87.3% in both revision sinus surgery and cases with distorted sinus anatomy of development, postoperative, or traumatic origin; 75.3% in cases with extensive sinonasal polyps; 80.4% in cases with pathology involving the frontal, posterior ethmoid, and sphenoid sinuses; 73.4% in cases with disease abutting the skull base, orbit, optic nerve or carotid artery; 63.9% in both CSF rhinorrhea or conditions where there is a skull base defect, and benign and/or malignant sino-nasal neoplasms (Fig. 12).

## Discussion

This study investigated the current availability and usage of IGS amongst Canadian otolaryngologists. The results of this study confirm that there are regional and institutional disparities regarding the use of IGS. Only 56% of respondents reported having access to IGS with 85% of those without IGS expressing interest in using it. This study sought to quantify and determine the reason for these discrepancies across the country with the hope to help inform surgeons, institutions, and policy makers. Understanding these discrepancies may help identify appropriate areas of need and future expansion of IGS.

The utility of IGS in sinus surgery has been well studied. A meta-analysis by Vreugdenburg et al. of 8 studies with over 800 procedures found a decrease of major, orbital, and total complications [7]. Another meta-analysis by Dalgorf also found that IGS significantly reduced complication rates in ESS [3]. IGS usage for ESS is also associated with more “complete” sinus surgery [8] leading to reduced surgical revision rates [9, 10]. There are currently no official Canadian guidelines for IGS indications. The American Academy of Otolaryngology–Head and Neck Surgery (AAO-HNS) have published a position statement regarding the use of IGS for select cases to help guide surgical decision-making [6]. Examples of indications in which the use of IGS may be deemed appropriate include the following: revision sinus surgery; distorted sinus anatomy of development; postoperative, or traumatic origin; extensive sino-nasal polyposis; pathology involving the frontal; posterior ethmoid and sphenoid sinuses; disease abutting the skull base, orbit, optic nerve or carotid artery; CSF rhinorrhea or conditions where there is a skull base defect; benign and malignant sino-nasal neoplasms. The lack of consensus and official Canadian guidelines may play a role in the discrepancy and variability of IGS usage found among different Canadian institutions. The issue of IGS availability in Canada however may not be an issue for American otolaryngologists whereby a 2010 mail IGS survey found that 94.7% of respondents had access to IGS [1].

Compared to our American colleagues, only 56% of our survey respondents reported having access to IGS with 85% of those without IGS expressing interest in using it. Moreover, 83.1% of these respondents mentioned that a lack of IGS access prevented them from performing surgeries they would otherwise undertake. Of these respondents, 48% are performing two to five routine ESS per month and 20% five to ten routine ESS per month. More importantly, 54% of these respondents are performing one to two complex/revision ESS per month which are generally regarded as indications for IGS. As can be expected, the significant majority of academic otolaryngologist reported access to IGS (90.3%) whereas only 40.6% of community otolaryngologist mentioned IGS access. The significance of this lack of access is further highlighted by additional comments written by some of the respondents. One respondent without IGS access mentioned “I would be able to better perform revision surgery and sphenoid sinus surgery” while another mentioned “I would be able to do more aggressive dissection in my polyp cases”. One respondent wrote “I routinely perform less complete FESS to err on the side of safety because of the lack of IGS. I also refer patients who have had very minimal previous sinus surgery, where I could easily do their cases, but because these are technically revision cases, medicolegally, I should not be doing them without IGS”. It is clear that a lack of IGS access is playing a role in limiting surgeon comfort during ESS. It is also limiting the amount of sinus surgery offered to patients especially with avoidance of frontal sinus surgery whereby respondents have mentioned they would perform more “complex frontal cases, revision cases”, other comments included “I’m not doing frontal sinus surgery without image guidance”, “I avoid frontal sinus surgery and revision sinus surgery”, “limited frontal sinus work”, and “it would be nice for revisions and difficult frontal sinus anatomy”. It is abundantly clear that a significant amount of Canadian otolaryngologists are being limited by lack of IGS in performing certain cases or are performing cases whereby IGS is indicated without it.

Regarding potential drawbacks of using IGS, capital cost and disposable cost were identified as the most important or major factors to IGS use (65.2% and 44.3% of respondents, respectively). Additional comments where made by respondents with concerns about equipment setup. One respondent mentioned “our facilities struggle with the setup and maintenance of the IGS” and another expressed a need for “nursing education on IGS equipment and setup”. Regarding potential benefits of using IGS, completeness of surgery and safety were identified as the most important or major factors of IGS use (86.7% of respondents for each factor, respectively). Overall, respondent comments for benefits of IGS were very favourable with comments such as “[IGS is] a must” and “I think most ENTs would want their sinus surgery done with IGS since in experienced hands it helps with patient safety and thoroughness of surgery”.

Despite widespread acceptance of IGS as an adjunctive tool for ESS, the accessibility of this technology remains variable across hospitals in Canada. This study demostrates that surgeons are limiting their surgical practice due to a lack of access to IGS. Most importantly, this affects the availability of surgical care to patients in Canada. We’ve also identified significant provincial disparity whereby 88% of British Columbia community otolaryngologist have IGS access and only 10% Quebec community otolaryngologist report access to IGS**.** One of the primary tenants of health care in Canada in equitable care, and the results of this study highlights a significant disparity in the ability of surgeons in Canada to provide equitable access to image guided sinus surgery. IGS is associated with a significant capital cost, and this was the most common reported reason for unavailability of IGS. However, the benefits of IGS have been well demonstrated, including improved patient safety and more complete surgery. The results of this study will help inform institutions identify the ongoing disparity of care. This study also demonstrates surgeons without access to IGS have a significant interest to in obtaining it. Hopefully, defining the need, variation and accessibility issues will help support surgeons in their pursuit to obtain IGS.

### Limitations

One potential limitation of this study is an inherent selection bias; respondants may have an interest in IGS, whereas those without an interest may not have responded. It was also unclear if all emails obtained from the society mailing lists were active or if any multiple contacts provided may have artificially lowered the response rate. The majority of the responses were from Ontario and Quebec. Unfortunately, we did not receive as many responses from the other provinces, most notably, Alberta, Manitoba and Saskatchewan with response rates of 7.0%, 4.4% and 2.5% respectively.

## Conclusion

Over half of Canadian otolaryngologists have access to IGS, with over 85% of those without access interested in using this technology if it was made available. Lack of access to IGS was identified as a barrier to performing certain surgical cases, and this may limit patient access to care across Canada. There is significant regional variation in access, which may lead to variability in care in sinonasal disease across Canada. The results of this study highlight the variation and disparity in access to IGS across Canada. As we strive to provide equitable and accessible care to patients, the results of this study will help inform physicians and their institutions of a need to improve access to IGS across Canada.

## Data Availability

Data supporting the results reported in the article can be obtained by contacting the corresponding author whereby a link to the google form can be shared upon request.
