# Correction: Image guidance system use amongst Canadian otolaryngologists: a nationwide survey

**DOI:** 10.1186/s40463-022-00589-3

**Published:** 2022-07-28

**Authors:** T. McHugh, D. D. Sommer, A. Thamboo, M. A. Tewfik, K. A. Smith

**Affiliations:** 1 Division of Otolaryngology, Head and Neck Surgery, Department of Surgery, McGill University, Montreal, QC Canada; 2 Division of Otolaryngology, Head and Neck Surgery, Department of Surgery, University of British Columbia, Vancouver, BC Canada; 3 Department of Otolaryngology, Head and Neck Surgery, University of Manitoba, Winnipeg, MB Canada; 4 ENT Clinic, The Montreal Children’s Hospital, 1001 Boulevard Décarie, A.RC.4221, Montréal, QC H4A 3J1 Canada

## Correction to: Journal of Otolaryngology - Head & Neck Surgery (2022) 51:27 https://doi.org/10.1186/s40463-022-00581-x

The original article contained errors in the presentation of the authors’ names which have since been corrected.
